# Effect of a prescriptive dietary intervention on psychological dimensions of eating behavior in obese adolescents

**DOI:** 10.1186/1479-5868-10-119

**Published:** 2013-10-24

**Authors:** Mandy Ho, Megan Gow, Jocelyn Halim, Kerryn Chisholm, Louise A Baur, Manny Noakes, Katherine Steinbeck, Michael R Kohn, Chris T Cowell, Sarah P Garnett

**Affiliations:** 1 The Children’s Hospital at Westmead Clinical School, University of Sydney, Locked Bag 4001, Westmead, NSW 2145, Australia; 2 Institute of Endocrinology and Diabetes, The Children’s Hospital at Westmead, Locked Bag 4001, Westmead, NSW 2145, Australia; 3 Kids Research Institute, The Children’s Hospital at Westmead, Locked Bag 4001, Westmead, NSW 2145, Australia; 4 Nutrition and Dietetics and Weight Management Services, The Children’s Hospital at Westmead, Locked Bag 4001, Westmead, NSW 2145, Australia; 5 CSIRO Food and Nutritional Sciences, PO Box 10041, Adelaide BC, SA 5000, Australia; 6 Academic Department of Adolescent Medicine, Sydney Medical School, University of Sydney, Sydney, NSW 2066, Australia; 7 Centre for Research into Adolescent’s Health, The Children’s Hospital at Westmead, Locked Bag 4001, Westmead, NSW 2145, Australia

**Keywords:** Eating behavior, Structured meal plan, Dietary intervention, Obesity, Adolescent

## Abstract

**Background:**

Overweight adolescents are more likely to have dysfunctional eating behaviours compared to normal weight adolescents. Little is known about the effects of obesity treatment on the psychological dimensions of eating behavior in this population.

**Objective:**

To examine the effects of a prescriptive dietary intervention on external eating (eating in response to food cues, regardless of hunger and satiety), emotional eating and dietary restraint and their relation to weight loss. Parental acceptability was also examined.

**Method:**

This is a secondary study of a 12-month randomized trial, the RESIST study, which examined the effects of two diets on insulin sensitivity. Participants were 109 obese 10- to 17-year-olds with clinical features of insulin resistance. The program commenced with a 3-month dietary intervention using a structured meal plan, with the addition of an exercise intervention in the next 3 months and followed by a 6 month maintenance period.This paper presents changes in eating behaviors measured by the Eating Pattern Inventory for Children and parent rated diet acceptability during the first 6 months of the trial. As there was no difference between the diets on outcome of interest, both diet groups were combined for analyses.

**Results:**

After 6 months, the proportion of participants who reported consuming more in response to external eating cues decreased from 17% to 5% (P = 0.003), whereas non- emotional eating increased from 48% to 65% (p = 0.014). Dietary restraint and parental pressure to eat remained unchanged. A reduction in external eating (rho = 0.36, P < 0.001) and a reduction in dietary restraint (r = 0.26, P = 0.013) were associated with greater weight loss at 3 and 6 months, respectively. Overall this approach was well accepted by parents with 72% of parents considered that their child would be able to follow the meal plan for the longer term.

**Conclusions:**

In the short to medium term, a prescriptive dietary intervention approach is a well-accepted and suitable option for obese adolescents with clinical features of insulin resistance. It may reduce external and emotional eating, led to modest weight loss and did not cause any adverse effect on dietary restraint.

**Trial registration:**

Australian New Zealand Clinical Trial Registration Number (ACTRN) 12608000416392 https://www.anzctr.org.au/Trial/Registration/TrialReview.aspx?id=83071

## Introduction

Dietary intervention as part of lifestyle intervention improves weight status and cardio-metabolic outcomes in overweight children and adolescents in the short to medium term [1]. However, the question of what dietary intervention approach is most effective in treating childhood obesity remains unanswered. There is evidence that obese adolescents seeking weight management have a preference for prescriptive dietary advice, as opposed to the current standard model of unstructured advice [2]. Studies in adults show that prescriptive dietary intervention approaches lead to greater weight loss and greater improvement in psychological outcomes compared with generalised lifestyle advice [3].

Obesity is due to a complex interplay of genetic, behavioral, social and environmental factors. Dysfunctional eating behaviors are posited to contribute to overweight [4,5]. These include external eating (eating in response to external food-cues, such as the sight and smell of food, regardless of the internal state of hunger and satiety), emotional eating (eating in response to negative emotions) and dietary restraint (cognitive determination and efforts to restrict food intake in order to control body weight). Overweight children are more likely to have dysfunctional eating behaviors compared to normal weight children [4-8] and high levels of external and emotional eating are, in the long term, associated with a decreased responsiveness to internal hunger and satiety [7]. Thus, these behaviours are hypothesized to be linked to overeating and overweight in the long term [4,7]. The impact of restrained eating on weight control is less clear. One view is that excessive restraint is a risk factor for overeating [9] and weight gain [4,10]. Others argue that dietary restraint can be a positive coping strategy for weight loss and weight maintenance [11,12]. Additionally, children’s eating behavior and weight status is linked to parents’ feeding practices [13]. Parental pressure to eat has been shown to have a disruptive effect on the self-regulating mechanism of food intake in children [8,14], causing children to stop using physiological satiety to terminate their eating.

In adult studies, dysfunctional eating behavior changes significantly after obesity treatment [15]. Little is known about the effects of obesity treatment on the psychological dimension of eating behavior in paediatric populations. The aims of this study were:

1. to examine the effects of implementing a structured meal plan on external eating, emotional eating, dietary restraint and perceived parental pressure to eat in adolescents with clinical features of insulin resistance;

2. to determine the relation between the change in eating behavior and weight loss;

3. to examine parental acceptability of the structured meal plan.

## Methods

### Participants

Participants were taking part in a 12 month randomised trial, the RESIST trial, examining the effect of two prescribed diets on insulin sensitivity in adolescents with clinical insulin resistance and/or prediabetes. The study protocol [16] and the primary outcomes after 6 months of intensive intervention [17] have been previously published. In brief, eligibility criteria were adolescents aged between 10 to 17 years at recruitment, who were overweight or obese [18], and with either prediabetes and/or clinical features of insulin resistance.

The study was approved by the Human Research Ethics Committee of The Children’s Hospital at Westmead (07/CHW/12), Sydney South West Area Health, Western Zone (08/LPOOL/195) and Sydney South West Area Health Service, Royal Prince Alfred Hospital (08/RPAH/455). The RESIST study recruited 111 participants between January 2009 and November 2011 through doctor referrals from three tertiary hospitals in Sydney. Written informed consent was sought from parents, and assent from participants, prior to study enrolment.

### Interventions

There were three phases: an intensive structured dietary intervention (Phase I, 0 to 3 months), an intensive exercise program plus ongoing dietary support (Phase II, 4 to 6 months), and a maintenance phase (Phase III, 7 to 12 months). All participants were treated with metformin and received the same overall lifestyle intervention. The only difference between the two intervention groups was the macronutrient content of the prescribed diets. This paper presents results related to Phases I and II.

#### Diet

The two prescribed diets were isocaloric and consisted of a moderate carbohydrate, increased protein diet (40-45% of total energy as carbohydrate, 30% fat and 25-30% protein) or a high carbohydrate diet (55-60% of total energy as carbohydrate, 30% fat and 15% protein). Both diets were prescriptive and two different energy levels were prescribed depending upon age: 6000 to 7000 kJ (10 to 14 year olds) or 7000 to 8000 kJ (15 to 17 year olds). Details of the delivery of the dietary intervention have been previously described [16]. Briefly, in Phase I the participant and at least one parent/carer attended four 60-minute face to face meetings (baseline, week 2, 6 and 12) with the trial dietician. The dietician delivered a standardised intervention to both intervention arms using a structured meal plan detailing the food choice and portion size for each main meal and snack. The meal plans took into consideration adolescent food preferences, with the aim of weight loss.

The trial dietician acted as a nutrition coach for the participating family. The coaching framework utilised a number of key psychological variables promoting self-efficacy, self-monitoring and goal setting in order to effect dietary compliance, lifestyle change and sustainable weight loss. For example, at each diet session participants set goals, developed a task list based on the mutually agreed goals, and were provided a dietary checklist for self-monitoring at home. In addition to the face to face contact, the dietician also contacted participants at weeks 4 and 9 using either phone, email or text message, to assist with motivation and answer participants’ questions. During Phase I food consistent with the prescribed diet plans and equating to approximately 25% of the participants’ energy requirements was provided to the families. During Phase II, participants received nutritional support (phone, email or SMS) every 4 weeks, with a face to face session at week 26.

All face to face sessions and phone/email/text message supports followed a standardized study protocol. Participants were encouraged to follow their prescribed diet for the duration of the trial. The meal plan was reviewed at each follow up session to ensure that there was enough variety and adequate amounts of food. Alternate food choices with similar energy and macronutrient contents were provided upon request. A snack food consisting of 700 kJ was added to the original meal plan if participants reported feeling hungry.

#### Exercise

At Phase I, participants received standardised physical activity advice delivered by the trial dietician. The advice was consistent with Australian recommendations for children and adolescents, including promoting an increase in incidental activity, a decrease in sedentary behavior and an increase in active transport [19]. During Phase II, all participants received a supervised exercise program for 45 to 60 minutes, twice a week for 12 weeks, in a commercial gym or a local park in the geographic area in which they lived.

### Measurements

#### Psychological dimensions of eating behaviors

Participants completed the Eating Pattern Inventory for Children (EPI-C) [20] at baseline, 3 and 6 months. EPI-C is a self-reporting tool which was originally modified from eating behaviour measures for adults and has been validated in 8–11 year old children [20]. It consists of 20-items and four subscales: external eating, emotional eating, dietary restraint and parental pressure to eat. Response choices were listed in a 4-point Likert scale format (1 = *not at all*, 2 = *sometimes*, 3 = *mostly*, 4 = *always*). Scores for each subscale were obtained by dividing the total scores by the total number of items in the respective subscale, with each subscale having a score ranging from 1 to 4. Higher scores in the respective subscales were indicative of greater external eating, emotional eating, dietary restraint or parental pressure to eat. The internal consistency (Cronbach’s α) for the present sample at baseline was 0.78, 0.80, 0.82 and 0.60 for external eating, emotional eating, dietary restraint and parental pressure to eat, respectively.

#### Diet acceptability

The parent/carer completed a locally developed 36-item dietary intervention questionnaire at the conclusion of both Phases I and II. The questionnaire aimed to understand how the prescribed diet affected the participants’ and their family’s patterns of living. The majority of test items had a 5-option response format: none, a little, some, most or all of the time. Parents also rated how easy and how pleasant the prescribed eating pattern was to follow on a 9-point scale (-4 indicating most difficult or most unpleasant, and +4 indicating most easy or most pleasant), and whether their child was able to follow the eating pattern long term (yes or no).

#### Anthropometry

Weight and height were measured using standard procedures as previously described [21]. Body mass index (BMI), expressed as a percentage of the 95th centile (BMI%95 centile), was calculated from age and sex specific reference values and used to measure the change in levels of adiposity [22]. Change in BMI z-score was not used as >96% of the adolescents had a BMI >97th centile which is beyond the scope of the CDC 2000 reference data [23].

### Statistical analysis

Data were assessed for normality and analysed using PASW statistical software for Windows, version 20 (SPSS Inc, Chicago, IL). Differences between continuous data were examined using independent sample t tests for normally distributed data, or Mann–Whitney tests for non-parametric data. Chi-squared tests were used to test for differences in categorical data and odds ratios were used to examine the magnitude of the association. McNemar’s Test was used to analyse paired categorical data to test for group differences and change over time. Correlations between variables were assessed by Pearson’s correlation coefficients or Spearman’s rho for normally distributed and non-parametric data, respectively. Consistent with an intention-to-treat approach, all available data for participants as originally randomly assigned, were retained. Linear mixed models with an unstructured covariance structure were used to test for the effects of diet and time (baseline, 3 months and 6 months). Models were adjusted for sex, age and BMI. Post-hoc tests were conducted using a Bonferonni adjusted method. Non-parametric data (external eating score) were log transformed. The assumptions of modelling were tested and met. The Friedman Test was conducted to examine the change of emotional eating scores over time because the data was highly skewed. Subgroup analysis was conducted comparing boys and girls. Statistical significance was accepted at a level of P < 0.05.

## Results

The primary dataset contained 111 participants (66 girls), but this article examines the data of the 109 who returned their EPI-C at baseline. Table 1 shows the baseline characteristics of the participants. The median age was 13.2 [range 10.1 to 17.4] years, most (n = 105) were obese, and the mean BMI was 34.1 (SD 5.4) kg/m^2^. Baseline characteristics, including the self-reported eating behavior and parental pressure to eat, were not significantly different between the two diet groups except that a higher proportion of participants in the high carbohydrate, low fat diet group came from a single parent family.

**Table 1:** **Baseline characteristics**^**1**^

	**Male**	**Female**	**Overall**
	**(n = 45)**	**(n = 64)**	**(n = 109)**
Age, years, median [range]	13.4 [10.6 to 16.4]	12.9 [10.1 to 17.4]	13.2 [10.1 to 17.4]
Obese^2^	45 (100)	60 (94)	105 (96.3)
Height, metre, mean ± SD	1.67 ± 0.12	1.60 ± 0.91	1.63 ± 0.11
Weight, kilograms, mean ± SD	96.9 ± 20.1	86.8 ± 19.1	91.0 ± 20.0
BMI, kg/m^2^, mean ± SD	34.5 ± 4.7	33.8 ± 6.0	34.1 ± 5.37
BMI% 95 centile, mean ± SD	137 ± 21	129 ± 21	132 ± 21
Parent highest education	(n = 45)	(n = 53)	(n = 98)
Year 10 or below	13 (29)	22 (42)	35 (36)
Completed Year 12	8 (18)	5 (9)	13 (13)
Technical school/tertiary education	24 (53)	26 (49)	50 (51)
Family income, AUD/year	(n = 43)	(n = 52)	(n = 95)
<$31200/year	14 (33)	14 (27)	28 (30)
$31200 – $67599/year	17 (40)	21 (40)	38 (40)
≥$ 67600/year	12 (28)	17 (33)	29 (31)
Single parent family	(n = 45)	(n = 53)	(n = 98)
	10 (22)	16 (30)	26 (27)
Parent country of birth	25 (24)	23 (39)	(n = 103)
Australia/New Zealand	9 (21)	14 (24)	34 (33)
America/Europe	12 (27)	10 (17)	23 (22)
Asia	8 (18)	10 (17)	22 (21)
Africa/Middle East	4 (9)	2 (3)	18 (18)
Pacific Island			6 (6)
Spoke another language at home	(n = 43)	(n = 63)	(n = 106)
	9 (21)	12 (19)	21 (20)

^1^Values are number of participants (%) unless otherwise indicated.

^2^Obesity were defined by the criteria of the International Obesity Taskforce (21).

Of the 109 participants who commenced the study, 91 completed the 6-month intervention and returned the 6 month EPI-C. Attrition was the same for both intervention arms. Completers and dropouts did not differ in age, sex, BMI, parental educational levels, family income, parental country of birth, nor their baseline eating behaviors scores and levels of parental pressure to eat. However, dropouts were more likely to come from a single parent family (odds ratio 4.1 [95%CI 1.3 to 12.9]).

Changes in eating behavior, parent rated diet acceptability, and weight loss were not statistically different between the two intervention groups over the 6 months of intervention. Both diet groups therefore were combined for analyses and reporting.

### External eating

**Figure 1:**
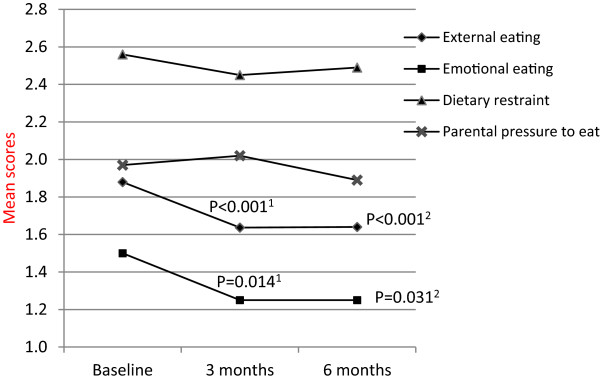
**Eating behaviours and parental pressure to eat at baseline, 3 and 6 months.** Estimated marginal means are presented from linear mixed models (♦ external eating, geometric means, ▲dietary restraint, **X** parental pressure to eat), and median presented from Wilcoxon Signed Rank Tests (■ emotional eating). ^1^ compared 3 months to baseline. ^2^ compared 6 months to baseline.

At baseline, 17% reported eating more in response to external cues either frequently or always (score ≥2.5) (Table 2). The baseline external eating score was positively correlated with baseline BMI%95 centile (rho = 0.19, P = 0.045) and emotional eating (rho = 0.46, P < 0.001). Age, sex, and family factors (including parental educational level, family income, family structure and parental country of birth) were not related to external eating at baseline. Intention-to-treat analysis found a significant decrease in external eating scores over 6 months in both groups. The geometric mean of external eating score was 15% less at 3 months (P < 0.001) and remained significantly lower from baseline at 6 months (P < 0.001) (Figure 1). Completer analysis showed the same results as intention-to-treat analysis. The proportion of participants who reported external eating frequently or always reduced from 17% to 2% at 3 months (P = 0.002) and was maintained at 5% at 6 months (P = 0.003). Overall, 80% of participants reported a reduction in external eating score at 6 months.

**Table 2:** The psychological dimensions of eating behavior at baseline, 3 and 6 months

		**%**^**1**^		
		**Baseline**	**3 months**	**6 months**
External eating *§	Not at all	23	36	42
	Sometimes	60	62	53
	Frequently/always	17	2	5
Emotional eating §	Not at all	48	59	65
	Sometimes	43	35	29
	Frequently/always	9	6	6
Dietary restraint	Not at all	2	5	2
	Sometimes	41	47	43
	Frequently/always	57	48	55
Parental pressure to eat	Not at all	32	37	37
	Sometimes	42	33	44
	Frequently/always	26	30	19

^1^Values are proportion of the 91 adolescents who completed the intervention and returned the eating behaviour questionnaires.

*significant difference between baseline and 3 months by the McNemar’s Test.

§significant difference between baseline and 6 months by the McNemar’s Test.

Although the self-reported external eating score was not significantly different between boys and girls at baseline, girls reported a greater reduction in external eating score compared to boys in the first 3 months (P = 0.005). Completer analysis revealed that the proportion of boys who reported external eating frequently or always (score ≥2.5) decreased from 23% at baseline to 7% at 3 months (P = 0.039) and was maintained at 11% at 6 months (P = 0.453), whereas that for girls decreased from 14% to none (0%) at 3 months and was maintained at 2% at 6 months.

### Emotional eating

At baseline 9% of participants reported using eating as a form of coping with emotional distress either frequently or always (score >2.5) while 48% reported not ever using eating as a form of coping with emotional stress (score <1.5) (Table 2). Similar to external eating, the baseline emotional eating score was positively correlated with baseline BMI%95 centile (rho = 0.21, P = 0.029) and was not related to age, sex, or family factors. There was a significant decrease in emotional eating scores over 6 months (P = 0.024). Post-hoc tests revealed a significant reduction in emotional eating scores from baseline to 3 months (P = 0.014) which was maintained lower at 6 months (P = 0.031) (Figure 1). Non-emotional eating increased from 48% at baseline to 65% at 6 months (P = 0.014). Change in emotional eating was not different between boys and girls.

### Dietary restraint

At baseline, 57% reported restrained eating either frequently or always (Table 2). No association between the dietary restraint score and participants’ age, sex, baseline weight status or family factors was found. Dietary restraint scores did not change over the 6 months (Figure 1).

### Parental pressure to eat

At baseline, 26% reported experiencing parental pressure to eat either frequently or always. Younger participants reported a higher level of parental pressure to eat (rho = -0.229, P = 0.017), and boys reported a higher parental pressure to eat score than girls (estimated marginal mean difference 0.302, P = 0.024). Baseline weight status and family factors were not related to perceived parental pressure to eat at baseline. Levels of parental pressure to eat remained unchanged over 6 months (Figure 1).

### Relationship between the change in self-reported eating pattern and weight change

Change in BMI%95 centile at 3 months was positively associated with change in external eating score (rho = 0.36, P < 0.001) and parental pressure to eat score (r = 0.20, P = 0.046), indicating that a decrease in external eating and a decrease in parental pressure to eat were related to greater weight loss, as determined by BMI%95 centile. At 3 months, 81 participants reported a decrease in, or maintained, their external eating score. Of these, 73 (90%) had a decrease in BMI%95 centile (median -7.9 [range -18.6 to -0.8]%).

However, change in BMI%95 centile at 6 months was only associated with change in dietary restraint score (r = 0.26, P = 0.013), indicating that reduced dietary restraint was associated with greater weight loss. Fifty-five participants had decreased or maintained their dietary restraint score at 6 months compared to baseline. Forty-seven of the 55 (86%) had a decrease in BMI%95 centiles (median -10.0 [range -38.0 to -0.1]%). Change in emotional eating score was not associated with weight change at any stage.

### Parent rated diet acceptability

**Figure 2:**
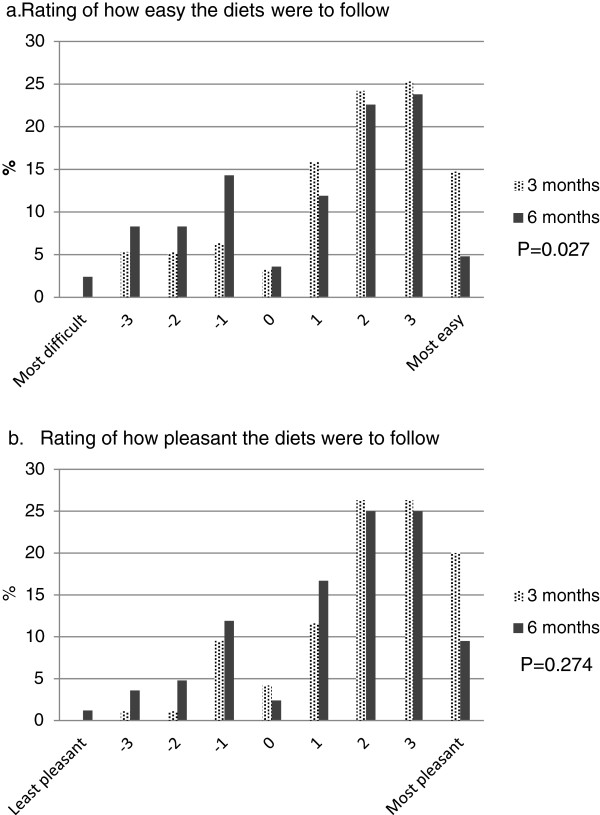
**Rating of easiness and pleasantness of the diets by parents at 3 and 6 months. a**. Rating of how easy the diets were to follow. **b**. Rating of how pleasant the diets were to follow. Parents rated how easy and how pleasant the prescribed eating pattern was to follow from a 9-point scale (-4 indicating most difficult or most unpleasant, and +4 indicating most easy or most pleasant).

At 3 months, most parents reported that their child felt good about the eating pattern (65%), felt more in control of their eating habits (67%) and was happy or content (75%) either most or all of the time (Table 3). Overall, 81% of parents thought that their child was able to follow the eating pattern long term. Diet acceptability remained satisfactory over 6 months, although a higher proportion of parents gave a neutral or negative rating for the ease of the prescribed meal plan to follow at 6 months (17% at 3 months vs 35% at 6 months, P = 0.027, Figure 2). Also, a higher proportion of parents felt that the eating pattern was difficult at school either most or all of the time at 6 months compared with that at 3 months (14% vs 4%, P = 0.021). No diet difference in the parent rated diet acceptability was found.

**Table 3:** Parent rated diet acceptability at 3 and 6 months

**Most or all of the time**	**%**^**1**^		**% change**^**2**^
	**3 months**	**6 months**	**(95% CI)**
Felt good about the eating pattern	67	58	9 (-33, 16)
Felt more in control of their eating habit	67	57	-10 (-34, 14)
Were happy or content	75	70	-5 (-31, 21)
Had enough food on the meal plan	79	76	-3 (-30, 25)
Did not follow the meal plan	9	10	1 (-9, 11)
Family members were positive about the eating pattern	88	80	-8 (-36, 21)
Meals were accepted by family members	86	75	-11 (-39, 16)
Some family members lost weight too	25	21	-4 (-19, 11)
Preparation of family meals was more difficult	4	7	3 (-4, 11)
The eating pattern was difficult at school*	4	14	10 (1, 19)
Family life was difficult for the child in the study	6	12	6 (-3, 16)
There were problems going out with family/friends	9	8	-1 (-10, 8)
Able to follow the eating pattern long term	81	72	-9 (-35, 18)
The study was beneficial for my child	89	83	-6 (-35, 22)

^1^Values are proportion of the 80 parents who completed the intervention and returned the diet acceptability questionnaires at 3 and 6 months.

^2^For change from the end of intensive dietary intervention phase (3 months) to 6 months.

*Significant change between 3 and 6 months by the McNemar’s Test.

As reported by parents, the prescriptive dietary intervention appeared to be more acceptable to girls than boys. According to parents’ report, a higher proportion of girls felt more in control of their eating habit most or all of the time compared with boys over the 6 months (at 3 months, girls 75%, boys 54%, P = 0.030; at 6 months, girls 64%, boy 41%, P = 0.036). Also, during Phase II, as reported by parents, 89% and 79% of girls in the study found enough food on the meal plan and were happy or content most or all of the time, respectively, whereas only 68% and 56% boys felt the same (P = 0.018 and 0.023, respectively). Furthermore, a higher proportion of parents with a daughter in the study considered the study to be beneficial to their child (91%) compared with those with a son (74%, P = 0.035).

### Relationship between diet acceptability and weight loss

At 3 months, participants whose parents reported that they felt more in control of their eating habits (odds ratio 2.6, [95%CI 1.0 to 6.9], P = 0.045) or that they could follow the meal plan long-term (odds ratio 3.3 [95%CI 1.3 to 8.4], P = 0.011) most or all of the time were about three times more likely to lose weight than their counterparts. However, no association between parents rated diet acceptability and level of weight loss at 6 months was found.

## Discussion

Understanding the psychological dimension of eating behavior in overweight children and adolescents is of clinical importance, as it could facilitate the effective tailoring of treatment to patient characteristics. This study demonstrated that, in the short to medium term, a structured and prescriptive dietary intervention approach is a suitable option for obese adolescents with clinical features of insulin resistance. This approach led a reduction in dysfunctional eating behaviors, particularly external and emotional eating and lead to modest weight loss.

Both external and emotional eating reduced significantly during the intensive dietary intervention phase (0 to 3 months) and levels were maintained to 6 months with continued weight loss. This finding is in agreement with those from a recent systematic review of adult studies [15]. One possible explanation is that the structured meal plan helped participants control the tendency to overeat to achieve the initial weight loss during the first 3 months, and facilitated other factors relevant to weight to support ongoing weight loss.

Different intervention approaches may lead to different treatment effects. People with high levels of external and emotional eating consume food not just because they are hungry but because they are controlled by external food cues or emotions [5]. A previous study of personality traits in obese adults demonstrated that external and emotional eating are associated with impulsiveness and lower self-control [24]. In addition, a study in 7 to 12 year olds has shown that emotional eating is positively associated with longer screen time which may pose a higher risk for mindless eating and the development of excess weight gain [25]. One important finding of the current study is that a prescriptive dietary intervention approach led to a reduction in emotional eating over 6 months. A structured and prescriptive meal plan with detailed instruction on types and portion size of food, as well as when to eat, may help to promote self-control skills, and therefore reduce the tendency for external and emotional eating. In our study, this speculation is supported by parents reporting that their child felt more in control of their eating habit and was happy or content during the study period. Therefore, a structured and prescriptive meal plan may be used as a coping strategy for external eaters to confront external food cues.

A reduction in dietary restraint score was associated with weight loss in the medium term (0 to 6 months). However, we are not able to draw any firm conclusions as to whether dietary restraint is a symptom, a cause or an effect of overweight. From both a theoretical and clinical point of view dietary restraint is to some extent required in order for weight control to occur. Nevertheless, according to the Theory of Restrained Eating, dietary restraint is a maladaptive behavior in obese people and is related to eating pathology via vicious cycles of eating, weight and shape concerns [9,26,27]. Excessive restraint may have a counterproductive effect and eventually lead to weight gain [4,10]. Consistent with the literature [5], we found that dietary restraint was prevalent among obese adolescents, as 60% of participants reported dietary restraint either frequently or always at baseline, with this remaining unchanged over the 6 months. This finding suggests that implementation of a prescriptive, low energy meal plan did not elicit further adverse effects on dietary restraint. However, additional studies are required to explore effective strategies for rectifying the dietary restraint traits in obese children and adolescents.

A high protein diet is considered to have a greater effect on satiety [28-30]. We had expected that the moderate carbohydrate, increased protein diet would elicit different effects on eating behaviors compared to the high carbohydrate and low fat diet. Nevertheless, there were no significant differences in the change in eating behaviors or diet acceptability between diet groups at any time point. Therefore, we speculate that the observed changes in external eating and emotional eating are due to the intervention approach rather than the macronutrient content of the diets. This preliminary finding should be confirmed with further research.

To our knowledge, this is the first study to report the effects of obesity treatment on the psychological dimensions of eating behaviors in adolescents and their relation to weight loss. There are several limitations in this study. Firstly, the questionnaire used in this study has been validated in pre-adolescents, whereas the participants of RESIST trial were 10 to 17 year olds. EPI-C was the instrument with best face validity for assessing the psychological dimensions of eating behaviors in children when the RESIST trial commenced. Of note, the internal consistency for the present sample was 0.8 for all the psychological dimensions of eating behaviors. Secondly, possible bias of self-reporting of eating behavior cannot be completely ruled out. Future studies may include parent reports of their child’s eating behavior as supplementary information. Thirdly, the possible effect on any outcomes of providing a proportion of food to the families in the first 3 months was not evaluated. In addition, as all participants in this study were prescribed metformin, the possible confounding effect of this medication on appetite control cannot be excluded. Furthermore, this paper is a secondary data analysis of an RCT examining the effects of two diets on the insulin sensitivity of obese adolescents with clinical features of insulin resistance. We did not have a control group who received no intervention and thus the findings should be interpreted with caution. Finally, as this study was conducted among obese adolescents with prediabetes and/or clinical features of insulin resistance, further research is needed to validate the effectiveness of the prescriptive dietary intervention approach compared with conventional lifestyle interventions in the general obese population.

In conclusion, in the short to medium term, a prescriptive dietary intervention approach has no adverse effect on the psychological dimensions of eating behaviors and was well-accepted by parents of obese adolescents with clinical features of insulin resistance. Due to the complex nature of obesity, different individuals may need different treatment approaches to achieve their weight loss goal. This study demonstrates that a prescriptive dietary intervention approach may be used as a coping strategy for external eaters. Further dietary intervention approaches for obese children with different eating styles, and the long-term effectiveness of prescriptive dietary interventions, need to be explored.

## Competing interest

The authors declare that they have no competing interest.

## Authors’ contribution

SPG, CTC, LAB and MN participated in all aspects of the conception and design of the study. KS and MKR participated in the design of the study. KC was responsible for the development of the dietary intervention and training of the trial dieticians. MG and JH conducted the study. MH performed data analyses and wrote the paper. MH and SG had full access to all the data in the study and take responsibility for the integrity of the data and the accuracy of the data analysis. All authors were involved in data interpretation and preparation of the manuscript and have read and approved the final manuscript.
